# Opinion formation in multiplex networks with general initial distributions

**DOI:** 10.1038/s41598-018-21054-0

**Published:** 2018-02-12

**Authors:** Chris G. Antonopoulos, Yilun Shang

**Affiliations:** 1 Department of Mathematical Sciences, University of Essex, Wivenhoe Park, UK; 2 School of Mathematical Sciences, Tongji University, Shanghai, China

## Abstract

We study opinion dynamics over multiplex networks where agents interact with bounded confidence. Namely, two neighbouring individuals exchange opinions and compromise if their opinions do not differ by more than a given threshold. In literature, agents are generally assumed to have a homogeneous confidence bound. Here, we study analytically and numerically opinion evolution over structured networks characterised by multiple layers with respective confidence thresholds and general initial opinion distributions. Through rigorous probability analysis, we show analytically the critical thresholds at which a phase transition takes place in the long-term consensus behaviour, over multiplex networks with some regularity conditions. Our results reveal the quantitative relation between the critical threshold and initial distribution. Further, our numerical simulations illustrate the consensus behaviour of the agents in network topologies including lattices and, small-world and scale-free networks, as well as for structure-dependent convergence parameters accommodating node heterogeneity. We find that the critical thresholds for consensus tend to agree with the predicted upper bounds in Theorems 4 and 5 in this paper. Finally, our results indicate that multiplexity hinders consensus formation when the initial opinion configuration is within a bounded range and, provide insight into information diffusion and social dynamics in multiplex systems modeled by networks.

## Introduction

The last decades witnessed many attempts to delineate the propagation of opinions or behaviours in a structured population by network science^1^, where individuals are located on the node set of a connected graph and characterised by their opinion. The study of opinion dynamics covers a wide range of topics of interest, such as collective decision-making, emergence of fads, minority opinion survival, and emergence of extremism, etc., in the communities of sociophysics, social simulation and complexity science. Varied models have been developed to explain how hierarchies^2^ and consensus^3–5^ may arise in a society. For more information and results in the broad field of social dynamics, we refer the reader to the comprehensive surveys in^6,7^.

Due to the striking analogy with spin systems, the opinion models with binary or discrete opinion space^3,7^ have dominated research in the Physics’ literature. In social contagion processes, however, when people having opinions toward something meet and discuss, they may adapt their opinions toward the other individual’s opinion and reach a compromise. In this context, continuous opinion space with opinions expressed in real numbers is more favourable since it allows adjustment in terms of averaging due to the continuous nature of the opinions. Examples include prices, tax rates or predictions about macroeconomic variables. Following this paradigm, a well-known continuous-opinion model has been proposed by Deffuant, Weisbuch, and others (Deffuant model)^8,9^, which further examines compromising agents under bounded confidence. In such models, an individual is only willing to take those opinions into account, which differ less than a certain bound of confidence *d* from their own. This assumption reflects the psychological concept of selective exposure, where people tend to avoid communication with those with conflicting opinions. Similar consideration has been adopted in the much studied Axelrod model for the dissemination of cultures^10^.

In the initial studies of Deffuant-type opinion models, agents in a network are assumed to be homogeneous and have the same confidence bound. For instance, it was shown in^8,11^ that there exists a universal critical confidence threshold *d*_*c*_ for the homogeneous Deffuant model, above which complete consensus is reached (namely, a single opinion cluster emerges) while below it, opinions diverge (namely, two or more opinion clusters are observed) through extensive simulations on complex networks, be them complete graphs, lattices, or scale-free networks. In recent years, agent-dependent multi-level confidence bounds have been incorporated into the model, which mirror the complicated physiological and psychological factors such as the disparity of people’s knowledge, experience, and personality; see e.g.^12–15^. The persuasion capacity of the mass media has also been found to play a role in opinion formation^16^. It is worth noting that most of them are based on numerical simulations with only a few exceptions^17–20^ due to the complicated nonlinear dynamics involved.

The opinion negotiation processes studied in the above works take place on networks containing edges of the same type and at the same temporal and topological scale. However, the real individuals in a society are usually simultaneously connected in multiple ways, which can make a non-additive effect on network dynamics^21,22^. People in a society, for example, interact through diverse relationships: friendship, partnership, kinship, vicinity, work-related acquaintanceship, to name just a few. Admittedly, a natural and more appropriate description of such systems can be given by using multiplex networks, where the networks are made up of different layers that contain the same nodes and a given type of edges in each layer. Some recent works have pointed out that multiplexity can result in intrinsically different dynamics from their equivalent single-layer counterparts. The irreducibility of the Ising and voter models on multiplex networks has been emphasized in^23,24^. Opinion competition dynamics on duplex networks has been studied in^25^, where coexistence of both opinions in the two layers has been found possible using mean-field approximation. In the context of culture dissemination, multiplexity is found to generate a qualitatively different dynamical behaviour for the Axelrod model, which produces a new stable regime of cultural diversity^26^. In addition to single information spreading process on multiplex networks, the coupling between different types of contact processes, such as opinion formation and disease spreading, has been investigated in the multiplex networks; see e.g.^27–29^. Synchronisation processes between different layers in multiplex networks featuring the interplay between distinctive topological structures and dynamics have been reported recently in^30,31^. An updated survey towards the spreading processes and opinion formation on multiplex networks can be found in^32^. To the best of our knowledge, little attention has been paid to the opinion evolution in the Deffuant model (featuring bounded confidence) in the context of multiplex networks. In^33^, the author first examined the Deffuant model in a multiplex network, which is modeled by an infinite line with multiple layers. The critical confidence threshold is analytically identified through probabilistic analysis and verified by numerical simulations.

In this paper, we aim to moving a step further in the direction of^33^ by considering both general initial opinion distributions and general multiplex networks. In the standard Deffuant model, the initial opinions are assumed to be independently and uniformly distributed in the interval [0, 1]. General initial distributions have been independently introduced in^19,34^. We first address the opinion formation with general initial distributions over 1-dimensional multiplex networks after introducing our model in the Model description section. We then generalise our results to higher-dimensional multiplex lattices and, to general multiplex networks satisfying some regularity conditions. We derive analytical expressions for the critical confidence bound, where both the structural multiplexity and the initial distribution play essential role. Interestingly, we show that multiplexity essentially impedes consensus formation in the situations when the initial opinion configuration is within a bounded range. On the other hand, if a substantial divergence exists in the initial opinions, whether it is bounded distributed or not, multiplexity is found to play no role in determining the critical confidence level. Extensive numerical simulations are provided with both constant and degree-dependent convergence parameters, and the paper is concluded with some open problems in the Discussion section.

## Methods

### Model description

The class of models considered here are examples of interacting particle systems^35^ combining features of multiplex networks. Given $\ell \in {\mathbb{N}}$, a multiplex network is a pair *G* = (*V*, *E*), made of $\ell$ layers *G*_1_, *G*_2_, …, ${G}_{\ell }$ such that each layer is a simple graph *G*_*i*_ = (*V*, *E*) with node set *V* and edge set ${E}_{i}\subseteq V\times V$ for I$i=\mathrm{1,}\ldots ,\ell$. Here, the node set *V* is shared by all layers and it can be either finite or infinite. The edge set of *G* consists of $\ell$ types of edges: $E={\cup }_{i=1}^{\ell }{E}_{i}$. From the perspective of graph theory, each edge between two nodes *u* and *v* in graph *G* is a multiple edge consisting of at most $\ell$ parallel edges, each of which belongs to a respective layer *G*_*i*_. We assume that each layer *G*_*i*_ has bounded degrees. Hence, each agent in the network *G* has a bounded number of neighbours and at most $\ell$ types of relationship. Without loss of generality, we may assume that the network *G* is connected since one could consider connected components separately in what follows.

In the Deffuant model^8,9^, two agents compromise according to the following rules: initially (at time *t* = 0), each agent $u\in V$ is assigned an opinion value ${X}_{0}(u)\in {\mathbb{R}}$ identically and independently distributed (i.i.d.) following some distribution ${\mathcal L} ({X}_{0})$. In the standard case, ${\mathcal L} ({X}_{0})$ is the uniform distribution over [0, 1]. Independently of this, in the *i*th layer, each edge $e\in {E}_{i}$ is independently assigned a Poisson process with rate *λp*_*i*_ with ${p}_{i}\in \mathrm{(0,1)}$ and *λ* > 0 for $i=1,\ldots ,\ell$. We assume that ${\sum }_{i=1}^{\ell }{p}_{i}=1$ without loss of generality. These Poisson processes defined on the edges in *E* govern the evolution of opinions. Specifically, let *X*_*t*_(*u*) be the opinion value of agent *u* at time *t* ≥ 0, which remains unchanged as long as no Poisson event happens for any edge in *E* incident to *u*. Let *d* > 0, *α*_1_ = 1 and ${\alpha }_{i}\in \mathrm{(0,}\,\mathrm{1)}$ for $i=2,\ldots ,\ell$. When at some time *t* the Poisson event occurs at an edge $e=\{u,v\}\in {E}_{i}$ for some *i*, such that the pre-meeting opinions of the two agents are ${X}_{t-}(u)\,:={\mathrm{lim}}_{s\to t-}{X}_{s}(u)$ and ${X}_{t-}(v)\,:={\mathrm{lim}}_{s\to t-}{X}_{s}(v)$, we set

1 $${X}_{t}(u)=\{\begin{array}{cc}{X}_{t-}(u)+\mu ({X}_{t-}(v)-{X}_{t-}(u)), & {\rm{if}}\,|{X}_{t-}(u)-{X}_{t-}(v)|\le {\alpha }_{i}d;\\ {X}_{t-}(u), & {\rm{otherwise}},\end{array}$$

and

2 $${X}_{t}(v)=\{\begin{array}{cc}{X}_{t-}(v)+\mu ({X}_{t-}(u)-{X}_{t-}(v)), & {\rm{if}}\,|{X}_{t-}(u)-{X}_{t-}(v)|\le {\alpha }_{i}d;\\ {X}_{t-}(v), & {\rm{otherwise}},\end{array}$$

where *μ* ∈ (0, 1/2) is the so-called convergence parameter. Therefore, if the two pre-meeting opinions lie at a distance less than a certain confidence bound from one another, the meeting agents will come closer to each other symmetrically, by a relative amount *μ*, where *μ* = 1/2 implies that the two agents meet halfway through. If not, then they will stay unchanged. It is worth noting that the model is well-defined since the bounded degree assumption ensures that almost surely (i.e., with probability 1) none of the Poisson events will be simultaneous for an infinite node set [35, p. 28].

The multiplexity in the above opinion model lies in two aspects. First, the interaction rates *λp*_*i*_ in each layer can be different. Second, the confidence bounds *α*_*i*_*d* in each layer can be different too. We might as well consider distinct convergence parameters *μ* = *μ*_*i*_ for the *i*th layer indicating different willingness to change one’s mind. However, it has been confirmed analytically and numerically that *μ* plays no role in the qualitative behaviour of the opinion dynamics; it rather only affects the convergence time^6,8,18,19^.

### Sharing a drink process

In the section, we briefly review the sharing a drink (SAD) process proposed in^18^, which is particularly useful in later analysis of the Deffuant model on ${\mathbb{Z}}$; see also^14,19,34^.

Let $k\in {\mathbb{N}}\cup \mathrm{\{0\}}$. The SAD process, denoted by ${\{{Y}_{k}(u)\}}_{u\in {\mathbb{Z}}}$, is a deterministic process defined iteratively as follows: set

3 $${Y}_{0}(u)=\{\begin{array}{ll}\mathrm{1,} & {\rm{for}}\,u=\mathrm{0;}\\ \mathrm{0,} & {\rm{for}}\,u\in {\mathbb{Z}}\backslash \mathrm{\{0\}.}\end{array}$$

For a given sequence of nodes ${u}_{1},{u}_{2},\ldots \in {\mathbb{Z}}$ and $\mu \in \mathrm{(0,}\,\mathrm{1/2]}$, we obtain the configuration ${\{{Y}_{k}(u)\}}_{u\in {\mathbb{Z}}}$ for $k\ge 1$ by setting

4 $${Y}_{k}(u)=\{\begin{array}{ll}{Y}_{k-1}(u)+\mu ({Y}_{k-1}(u+\mathrm{1)}-{Y}_{k-1}(u)), & {\rm{for}}\,u={u}_{k};\\ {Y}_{k-1}(u)+\mu ({Y}_{k-1}(u-\mathrm{1)}-{Y}_{k-1}(u)), & {\rm{for}}\,u={u}_{k}+\mathrm{1;}\\ \quad \quad \quad \quad \quad {Y}_{k-1}(u), & {\rm{for}}\,u\in {\mathbb{Z}}\backslash \{{u}_{k},{u}_{k}+\mathrm{1\}.}\end{array}$$

This procedure can be envisioned as a liquid-exchanging process on ${\mathbb{Z}}$. A glass is put at each site $u\in {\mathbb{Z}}$. At *k* = 0 only the glass located at the origin is full (with value 1) while all others are empty (with value 0). At each subsequent time step *k*, one picks two neighbouring glasses at *u*_*k*_ and *u*_*k*_ + 1, and pouring liquids from the glass with higher level to that with lower level by a relative amount *μ*. This gives rise to the SAD process. The following lemma on unimodality can be easily proved.

#### Lemma 1.

(Unimodality) *If u*_*j*_ ≠ −1 *for j* = 1, …, *k*, then ${Y}_{k}\mathrm{(0)}\ge {Y}_{k}\mathrm{(1)}\ge {Y}_{k}\mathrm{(2)}\ge \ldots$.

Fix $t > 0$ and consider the opinion model on ${\mathbb{Z}}$. Note that there exists a finite interval $[{u}_{\alpha },{u}_{\beta }]\subseteq {\mathbb{Z}}$ containing 0 such that the Poisson events on the boundary edges $\{{u}_{\alpha }-\mathrm{1,}\,{u}_{\alpha }\}\in {E}_{i}$ and $\{{u}_{\beta },{u}_{\beta }+\mathrm{1\}}\in {E}_{i}$ for all $i=1,\ldots ,\ell$ have not happened yet up to time *t*. Let *N* be the number of opinion adjustments that occur in $[{u}_{\alpha },{u}_{\beta }]$ up to time *t*. The times of these adjustments are arranged in the chronological order

5 $${\tau }_{N+1}\,:=0 < {\tau }_{N} < {\tau }_{N-1} < \ldots  < {\tau }_{1}\le t,$$

where we set ${\tau }_{N+1}\,:=0$ for convenience. For $k=1,\ldots ,N$, we write *u*_*k*_ as the left endpoint of the edge $\{{u}_{k},{u}_{k}+\mathrm{1\}}$ for which *u*_*k*_ and ${u}_{k}+1$ adjust opinions at time ${\tau }_{k}$. Given the sequence ${u}_{1},\ldots ,{u}_{N}$ (in this order) and $\mu \in \mathrm{(0,}\,\mathrm{1/2]}$, we obtain a SAD process ${\{{Y}_{k}(u)\}}_{u\in {\mathbb{Z}}}$ as defined by (3) and (4).

#### Lemma 2.

(Linear representation) *For* $k=0,1,\ldots ,N$,

6 $${X}_{t}\mathrm{(0)}=\sum _{u\in {\mathbb{Z}}}{Y}_{k}(u){X}_{{\tau }_{k+1}}(u\mathrm{).}$$

*Particularly*, $${X}_{t}\mathrm{(0)}=\sum _{u\in {\mathbb{Z}}}\,{Y}_{N}(u){X}_{0}(u)\,:=\sum _{u\in {\mathbb{Z}}}\,{Y}_{t}(u){X}_{0}(u)$$.

This lemma implies that the constructed SAD process resembles the dynamics of the corresponding Deffuant model backwards in time so that the state *X*_*t*_(0) in the model at any time *t* > 0 can be expressed as a weighted average of states at time 0 with weights given by an SAD configuration. See^18,33^ for a proof.

### Data availability

All data are generated by numerical simulations and they have all been reported in the paper.

## Results

### Opinion dynamics in 1-dimensional multiplex networks

In this section, we will consider the multiplex opinion model on the integers ${\mathbb{Z}}$, focusing on the general initial opinion distributions. More specifically, we take $G=(V,E)$ with $V={\mathbb{Z}}$ and ${E}_{i}=\{\{u,u+\mathrm{1\}}\,:\,u\in {\mathbb{Z}}\}$ for $i=1,\ldots ,\ell$. When $\ell =1$, *G* becomes a simplex network with only one type of edges. For this interaction network, the critical confidence threshold for opinion formation with i.i.d. uniform initial distribution in [0, 1] is ${d}_{c}=\mathrm{1/2}$^17,18^ and later extended to the multiplex 1-dimensional networks in^33^.

**Figure 1:**
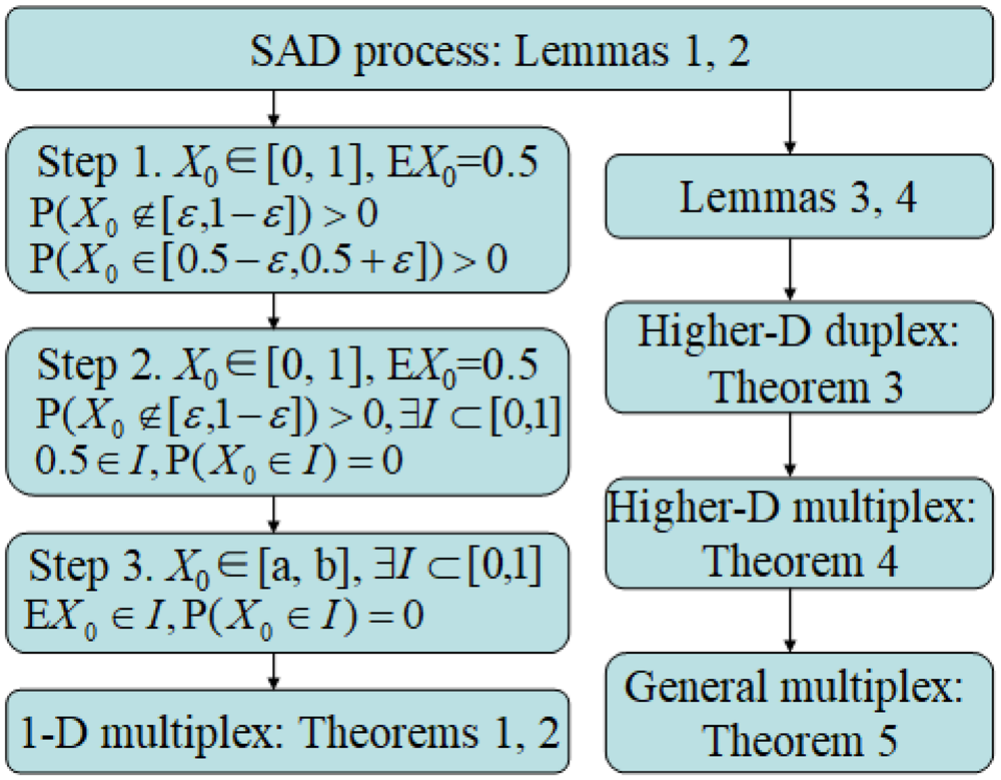
Schematic illustration of the theoretical results in the paper.

To appreciate this, we first present the results for the case $\ell =2$ (see Theorem 1) and then extend it to the general multiplex case (see Theorem 2), as illustrated in Fig. 1. To this end, we take $\ell =2$, $p={p}_{1}$, and $\alpha ={\alpha }_{2}$. With these assumptions, the main result concerning the critical confidence threshold for the 1-dimensional duplex model reads as follows.

#### Theorem 1.

(1-dimensional duplex networks) *Consider the above continuous opinion model (*$\ell =2$*) on* ${\mathbb{Z}}$ *with parameters* $\lambda ,d > 0$, $\mu \in \mathrm{(0},\mathrm{1/2]}$, and $\alpha ,p\in \mathrm{(0,}\,\mathrm{1)}$ *with α* > *μ*.

- *Suppose that the initial opinion follows some bounded distribution* ${\mathcal L} ({X}_{0})$ *with expected value* $E({X}_{0})$*, whose support is contained in the smallest closed interval [a, b]. Let* $h\ge 0$ *be the length of some maximal open interval* $I\subset [a,b]$ *satisfying* ${\rm{E}}({X}_{0})\in I$ *and* ${\rm{P}}({X}_{0}\in I)=0$*. Then*, ${d}_{c}=\,{\rm{\max }}\,\{({\rm{E}}{X}_{0}-a)(p+\alpha \mathrm{(1}-p{))}^{-1}$, $(b-{\rm{E}}{X}_{0})(p+\alpha \mathrm{(1}-p{))}^{-1},h\}$ *is the critical confidence threshold in the following sense*:
  *If* ${\rm{d}} < \,{\rm{\min }}\,\{{d}_{c},b-a\}$*, then with probability 1, there will be (infinitely many) finally blocked edges, namely*, $e=\{u,u+\mathrm{1\}}$ *satisfies* $|{X}_{t}(u)-{X}_{t}(u+\mathrm{1)| > }d$ *for all t large enough*;
  *If* ${\rm{d}} > \,{\rm{\min }}\,\{{d}_{c},b-a\}$*, then with probability 1*, ${X}_{\infty }(u)\,:={\mathrm{lim}}_{t\to \infty }{X}_{t}(u)={\rm{E}}({X}_{0})$ *for every* $u\in {\mathbb{Z}}$.
- *Suppose that the initial opinion distribution* ${\mathcal L} ({X}_{0})$ *is unbounded but its expectation exists in the sense of* ${\rm{E}}({X}_{0})\in {\mathbb{R}}\cup \{\pm \infty \}$*. Then, for any d* > *0, with probability 1, there will be (infinitely many) finally blocked edges, namely*, $e=\{u,u+\mathrm{1\}}$ *satisfying* $|{X}_{t}(u)-{X}_{t}(u+\mathrm{1)|}\, > \,d$ *for all t large enough*.

Before proceeding with the proof, we provide a couple of remarks. Firstly, when the initial distribution ${\mathcal L} ({X}_{0})$ is bounded and $d < \,{\rm{\min }}\,\{{d}_{c},b-a\}$, we will show that $\{|{X}_{t}(u)-{X}_{t}(u+\mathrm{1)|\}}\in \mathrm{\{0\}}\cup [d,b-a]$ for sufficiently large *t* and all $u\in {\mathbb{Z}}$, and hence, the integers split into (infinitely many) finite clusters of neighbouring agents asymptotically agreeing with each other, with no global consensus achieved. Secondly, in the special case of ${\mathcal L} ({X}_{0})$ being the standard uniform distribution in [0, 1], we readily reproduce Theorem 1 in^33^. A general ${\mathcal L} ({X}_{0})$ has been considered both theoretically and via simulations in^19,34^ for simplex networks (i.e., $\ell =1$). Theorem 1 can be thought of as an extension to multiplex networks. Finally, the assumption $\alpha  > \mu$ is required here for technical reasons as in^33^, which does not have a counterpart in the case of simplex networks where *μ* only influences the convergence time of the negotiation process.

The crucial technique adopted here is the SAD process introduced in^18^. The SAD process and its basic properties are briefly reviewed in the Method section. Another key concept from that paper is the flat-points concept. To accommodate the general distributions considered in the present paper, a slight extension of the definitions therein can be provided as follows. Given $\varepsilon  > 0$ and the initial opinion configuration ${\{{X}_{0}(v)\}}_{v\in {\mathbb{Z}}}$ with finite expectation, a node $u\in {\mathbb{Z}}$ is said to be an *ε-flat point to the right* if for all $n\ge 0$,

7 $$\frac{1}{n+1}\sum _{v=u}^{u+n}{X}_{0}(v)\in [{\rm{E}}({X}_{0})-\varepsilon ,{\rm{E}}({X}_{0})+\varepsilon ]\mathrm{.}$$

Likewise, $u\in {\mathbb{Z}}$ is said to be an *ε-flat point to the left* if for all $n\ge 0$,

8 $$\frac{1}{n+1}\sum _{v=u-n}^{u}{X}_{0}(v)\in [{\rm{E}}({X}_{0})-\varepsilon ,{\rm{E}}({X}_{0})+\varepsilon ],$$

and *two-sided ε-flat point* if for all $n,m\ge 0$,

9 $$\frac{1}{n+m+1}\sum _{v=u-n}^{u+m}{X}_{0}(v)\in [{\rm{E}}({X}_{0})-\varepsilon ,{\rm{E}}({X}_{0})+\varepsilon ]\mathrm{.}$$

We also define that $$u\in {\mathbb{Z}}$$ is an *ε-flat point to the right at time t* if for all $$n\ge 0$$, $$\frac{1}{n+1}{\sum }_{v=u}^{u+n}$$ $${X}_{t}(v)\in $$$$[{\rm{E}}({X}_{0})-\varepsilon ,{\rm{E}}({X}_{0})+\varepsilon ]$$. Similar definitions for *ε*-*flat point to the left at time t* and *two-sided ε-flat point at time t* can be given.

#### Proof of Theorem 1.

(a) Along the lines in [34, Section 2], we divide the proof of statement (a) into three steps.

*Step 1*. Suppose that the initial opinion distribution ${\mathcal L} ({X}_{0})$ is confined in [0,1] with expected value ${\rm{E}}({X}_{0})=\mathrm{1/2}$. Moreover, for any $\varepsilon  > 0$, we assume that ${\rm{P}}({X}_{0}\notin [\varepsilon \mathrm{,1}-\varepsilon ]) > 0$ and ${\rm{P}}\mathrm{(1/2}-\varepsilon \le {X}_{0}\le \mathrm{1/2}+\varepsilon ) > 0$ hold. Then we claim that ${{\rm{d}}}_{{\rm{c}}}={\mathrm{[2(}p+\alpha \mathrm{(1}-p))]}^{-1}$ is the critical confidence threshold in the same sense as in Theorem 1(a) (with $a=0$ and $b=1$).

To prove this claim, we need to show that the essential ingredients in the proof of Theorem 1 in^33^ still hold true. We mention here an obvious correction that the critical threshold separating the subcritical and supercritical regimes therein should be $${\rm{\min }}\,\{{d}_{c}\mathrm{,1\}}$$ instead of *d*_*c*_. For the subcritical regime, note that the fact that the mass is around the expected value, i.e., $${\rm{P}}\mathrm{(1/2}-\varepsilon \le {X}_{0}\le \mathrm{1/2}+\varepsilon ) > 0$$, implies that $${\rm{P}}(u\,{\rm{is}}\,\varepsilon -{\rm{flat}}\,{\rm{to}}\,{\rm{the}}\,{\rm{right}})=$$$${\rm{P}}(u\,{\rm{is}}\,\varepsilon -{\rm{flat}}\,{\rm{to}}\,{\rm{the}}\,{\rm{left}})$$ >0 for all $$\varepsilon  > 0$$ and $$u\in {\mathbb{Z}}$$ by similarly applying the coupling trick and the strong law of large numbers. At time *t* when a Poisson event occurs, define a Boolean random variable *A*_*t*_ by *A*_*t*_ = 1 with probability *p* and $${A}_{t}=\alpha $$ with probability 1 − *p* so that the opinion model constitutes a marked Poisson process with rate *λ*^33^. We can then mimic the proof for Propositions 1 and 2 in^33^ verbatim, which employs the condition $${\rm{P}}({X}_{0}\notin [\varepsilon ,\,1-\varepsilon ]) > 0$$ for any $$\varepsilon  > 0$$.

For the supercritical regime, we need to note that the property P(*u* is two-sided *ε*-flat) > 0 for any $\varepsilon  > 0$ and $u\in {\mathbb{Z}}$ can now be established by keeping in mind that ${\rm{P}}\mathrm{(1/2}-\varepsilon \le {X}_{0}\le \mathrm{1/2}+\varepsilon ) > 0$ following the same reasoning as in^18^; see also^34^. Now the proof for the supercritical regime in^33^ can be used, which concludes the proof of *Step 1*.

*Step 2*. Suppose that the initial opinion distribution ${\mathcal L} ({X}_{0})$ is again confined in [0,1] with expected value ${\rm{E}}({X}_{0})=\mathrm{1/2}$. For any $\varepsilon  > 0$, as in *Step 1* we assume that ${\rm{P}}({X}_{0}\notin [\varepsilon \mathrm{,1}-\varepsilon ]) > 0$. Moreover, assume that there exists some maximal open interval $I\subset \mathrm{[0},\mathrm{1]}$ of length *h* satisfying $\mathrm{1/2}\in I$ and ${\rm{P}}({X}_{0}\in I)=0$. Then, we claim that ${d}_{c}=\,{\rm{\max }}\,\mathrm{\{[2(}p+\alpha \mathrm{(1}-p{))]}^{-1},h\}$ is the critical confidence threshold in the same sense of Theorem 1(a) (with *a* = 0 and *b* = 1).

When $d < h$, thanks to the assumption ${\rm{P}}({X}_{0}\notin [\varepsilon \mathrm{,1}-\varepsilon ]) > 0$, we have initial opinions both below and above 1/2 with probability 1. Therefore, any edges which are blocked due to initial incident opinions lying on different sides of the gap *I* will remain blocked for all *t*. By ergodicity, there will be infinitely many such blocked edges, and thus consensus can not be reached in this case.

When $d > h$, we need to show that

10 $${\rm{P}}(u\,{\rm{is}}\,\varepsilon -{\rm{flat}}\,{\rm{to}}\,{\rm{the}}\,{\rm{right}}\,{\rm{at}}\,{\rm{time}}\,t)={\rm{P}}(u\,{\rm{is}}\,\varepsilon -{\rm{flat}}\,{\rm{to}}\,{\rm{the}}\,{\rm{left}}\,{\rm{at}}\,{\rm{time}}\,t) > 0$$

for all $\varepsilon  > 0$, $u\in {\mathbb{Z}}$ and for some sufficiently large *t*, since an arbitrary flat point at time *t* = 0 no longer exists due to the gap. Following the reasoning of [34, Section 2], one can then establish Eq. (10). The only minor change that has to be made in order to accommodate the multiplexity is that the involved marked Poisson processes has rate $\lambda p+\lambda \mathrm{(1}-p)=\lambda$ instead of a unit rate, which does not affect the validity of the proof. Now, as in *Step 1*, we can mimic the proof of Propositions 1 and 2 in^33^ verbatim to settle the subcritical case. Accordingly, ${d}_{c}\ge \,{\rm{\max }}\,\mathrm{\{[2(}p+\alpha \mathrm{(1}-p{))]}^{-1},h\}$. Next, the two-sided *ε*-flatness at time *t* for any *ε* > 0 can be established similarly as in [34, Section 2]. Hence, the argument in the supercritical case in *Step 1* leads to ${d}_{c}=\,{\rm{\max }}\,\mathrm{\{[2(}p+\alpha \mathrm{(1}-p{))]}^{-1},h\}$, completing the proof of *Step 2*.

*Step 3*. Now, everything is in place to prove Theorem 1(a) in its full generality.

Define $c\,:=\,{\rm{\max }}\,\{{\rm{E}}{X}_{0}-a,b-E{X}_{0}\}$ and perform the linear transformation $x\mapsto (x-{\rm{E}}{X}_{0}\mathrm{)/2}c+\mathrm{1/2}$ to the dynamics of our multiplex Deffuant model. Using the result in *Step 2* and the fact that the dynamics stays unchanged with respect to translations of the initial distribution and that parameter *d* can be re-scaled as per a scaling transformation of the initial distribution in order to recover the identical dynamics, we have

11 $$\begin{array}{rcl}{d}_{c} & = & 2c\,{\rm{\max }}\,\mathrm{\{[2(}p+\alpha \mathrm{(1}-p{))]}^{-1},h\mathrm{/2}c\}\\  & = & {\rm{\max }}\,\{({\rm{E}}{X}_{0}-a)(p+\alpha \mathrm{(1}-p{))}^{-1},(b-{\rm{E}}{X}_{0})(p+\alpha \mathrm{(1}-p{))}^{-1},h\mathrm{\}.}\end{array}$$

One can see that the ultimate consensus value in the supercritical regime is transformed from 1/2 to E*X*_0_ in view of *Step 2*.

(b) In the case of unbounded ${\mathcal L} ({X}_{0})$, we divide the proof into two cases.

*Case 1*. $${\rm{E}}|{X}_{0}| < \infty $$.

The strong law of large numbers implies that

12 $$P(\frac{1}{n+1}\sum _{v=u}^{u+n}{X}_{0}(v)={\rm{E}}{X}_{0})=1$$

for any $u\in {\mathbb{Z}}$. A simple calculation shows that node *u* is *δ*-flat to the right with positive probability for some $\delta  > 0$.

Fix *d* > 0. Following the reasoning in [33, Proposition 1] and noting that ${A}_{t}d\le d$, we can show that if *u* − 1 and *u* + 1 are *δ*-flat to the left and right respectively and ${X}_{0}(u)\notin [{\rm{E}}{X}_{0}-\delta -d,{\rm{E}}{X}_{0}+\delta +d]$ (which happens with positive probability), then ${X}_{t}(u-\mathrm{1)}$ and ${X}_{t}(u+\mathrm{1)}$ will stay in the interval $[{\rm{E}}{X}_{0}-\delta ,{\rm{E}}{X}_{0}+\delta ]$ for all *t* leaving the two edges $\{u-\mathrm{1,}\,u\}$ and $\{u,u+\mathrm{1\}}$ finally blocked. Since this event happens at each $u\in {\mathbb{Z}}$ with positive probability, it happens with probability 1 at infinitely many nodes by ergodicity.

*Case 2*. $${\rm{E}}{X}_{0}\in \{\pm \infty \}$$.

Without loss of generality, we assume that ${\rm{E}}{X}_{0}^{+}=\infty$ and ${\rm{E}}{X}_{0}^{-} < \infty$, where ${X}_{0}^{+}$ and ${X}_{0}^{-}$ are the positive and negative parts of *X*_0_, respectively. We may further assume that ${\rm{P}}({X}_{0}\le \mathrm{0)} > 0$, otherwise a translation would transform the problem to this case (c.f. *Step 3* above).

Fix *d* > 0. The same argument in [34, Section 2] can be used to show that the event $$\varepsilon \,:=\mathrm{\{(1/}n){\sum }_{v=u+1}^{u+n}$$$${X}_{0}(v) > d,{\rm{for}}\,{\rm{all}}\,n\in {\mathbb{Z}}\}$$ for any $$u\in {\mathbb{Z}}$$ happens with positive probability. Similarly, along the lines of [33, Proposition 1], we obtain that if *ε* happens and $${X}_{0}(u)\le 0$$ (which happens with positive probability), then $${X}_{0}(u+\mathrm{1)} > d$$ for all *t*. Namely, there will never be an opportunity for node *u* + 1 to average with *u*. The same thing holds for *u* − 1 by symmetry. Since the initial opinions are i.i.d., with positive probability we have $${X}_{0}(u)\le 0$$ and $${X}_{0}(u-\mathrm{1),}\,{X}_{0}(u+\mathrm{1)} > d$$, leaving the edges $$\{u-\mathrm{1,}\,u\}$$ and $$\{u,u+\mathrm{1\}}$$ finally blocked. Since this happens at every $$u\in {\mathbb{Z}}$$ with positive probability, by ergodicity, it happens with probability 1 at infinitely many nodes.□

For a multiplex network ${\mathbb{Z}}$ with $\ell$ layers, Theorem 2 is within easy reach by essentially using the same arguments as above.

#### Theorem 2.

(1-dimensional multiplex networks) *Consider the above continuous opinion model on* ${\mathbb{Z}}$ *with parameters* $\lambda ,d > 0$, $\mu \in \mathrm{(0},\mathrm{1/2]}$, and ${p}_{i}\in \mathrm{(0,}\,\mathrm{1)}$ for $i=1,\ldots ,\ell$, ${\alpha }_{i}\in \mathrm{(0,}\,\mathrm{1)}$ for $i=2,\ldots ,\ell$ and ${\alpha }_{1}=1$ with ${\alpha }_{i} > \mu$ for all *i*.

- *Suppose that the initial opinion follows some bounded distribution* ${\mathcal L} ({X}_{0})$ *with expected value* ${\rm{E}}({X}_{0})$*, whose support is contained in the smallest closed interval [a, b]. Let* $h\ge 0$ *be the length of some maximal open interval* $I\subset [a,b]$ *satisfying* ${\rm{E}}({X}_{0})\in I$ *and* ${\rm{P}}({X}_{0}\in I)=0$*. Then*, ${d}_{c}=\,{\rm{\max }}\,\{(E{X}_{0}-a)({\sum }_{i=1}^{\ell }\,{p}_{i}{\alpha }_{i}{)}^{-1},(b-{\rm{E}}{X}_{0})({\sum }_{i=1}^{\ell }\,{p}_{i}{\alpha }_{i}{)}^{-1},h\}$ *is the critical confidence threshold in the following sense*:
  *If* $d < {\rm{\min }}\{{d}_{c},b-a\}$*, then with probability 1, there will be (infinitely many) finally blocked edges, namely*, $e=\{u,u+\mathrm{1\}}$ *satisfies* $|{X}_{t}(u)-{X}_{t}(u+\mathrm{1)| > }d$ *for all t large enough*;
  *If* $d > \,{\rm{\min }}\,\{{d}_{c},b-a\}$*, then with probability 1*, ${X}_{\infty }(u):={\mathrm{lim}}_{t\to \infty }{X}_{t}(u)={\rm{E}}({X}_{0})$ *for every* $u\in {\mathbb{Z}}$.
- *Suppose that the initial opinion distribution* ${\mathcal L} ({X}_{0})$ *is unbounded but its expectation exists in the sense of* ${\rm{E}}({X}_{0})\in {\mathbb{R}}\cup \{\pm \infty \}$*. Then, for any* $d > 0$*, with probability 1, there will be (infinitely many) finally blocked edges, namely*, $e=\{u,u+\mathrm{1\}}$ *satisfies* $|{X}_{t}(u)-{X}_{t}(u+\mathrm{1)|} > d$ *for all t large enough*.

Several observations can be drawn from Theorem 2. Firstly, when the initial opinion distribution ${\mathcal L} ({X}_{0})$ follows the standard uniform distribution in [0, 1], we recover the previous result [33, Theorem 2]. Secondly, when ${\mathcal L} ({X}_{0})$ is bounded, since ${\sum }_{i=1}^{\ell }{p}_{i}=1$, we always have ${d}_{c}\ge \,{\rm{\max }}\,\{({\rm{E}}{X}_{0}-a),(b-{\rm{E}}{X}_{0}),h\}$, where the equality holds if and only if $\ell =1$ or $h\ge \,{\rm{\max }}\,\{({\rm{E}}{X}_{0}-a)({\sum }_{i\mathrm{=1}}^{\ell }\,{p}_{i}{\alpha }_{i}{)}^{-1},(b-{\rm{E}}{X}_{0})({\sum }_{i\mathrm{=1}}^{\ell }\,{p}_{i}{\alpha }_{i}{)}^{-1}\}$. This indicates it is more difficult to reach agreement over multiplex networks than simplex networks in general. When there is a large *h*, the critical confidence threshold *d*_*c*_ is dominated by *h* and is independent from the multiplexity; on the other hand, for relatively small *h*, the threshold is determined in turn by both the multiplexity and the initial distribution. When the initial distribution ${\mathcal L} ({X}_{0})$ is unbounded, consensus cannot be reached regardless of the multiplexity. Thirdly, if there exists some *k* satisfying ${p}_{k}\gg {p}_{j}$ for all $j\ne k$, then ${d}_{c}\approx \,{\rm{\max }}\,\{({\rm{E}}{X}_{0}-a){\alpha }_{k}^{-1},(b-{\rm{E}}{X}_{0}){\alpha }_{k}^{-1},h\}$ in the case of bounded ${\mathcal L} ({X}_{0})$. This suggests that the critical confidence is governed by a frequently interacted layer in the underlying network as one would expect.

### Opinion dynamics in general multiplex networks

In this section, we deal with more general multiplex networks and adopt a similar strategy by first looking into a duplex model on higher-dimensional lattices, generalising it to multiplex models and discussig on further extensions.

Particularly, we take $G=(V,E)$ with $V={{\mathbb{Z}}}^{D}$ for $D\ge 2$ and *E*_*i*_ consists of all edges in the *D*-dimensional lattice for $i=1,\ldots ,\ell$. When $\ell =1$, *G* becomes a simplex network with only one type of edges; see [34, Section 3]. For $\ell =2$, we denote $p={p}_{1}$ and $\alpha ={\alpha }_{2}$ as in the above section. The main result in this duplex case reads as follows.

#### Theorem 3.

(higher-dimensional duplex networks) *Consider the above continuous opinion model* ($\ell =2$) *on* ${{\mathbb{Z}}}^{D}$ *with* $D\ge 2$, $\lambda  > 0$, $\mu \in \mathrm{(0,}\,\mathrm{1/2]}$, and $\alpha ,p\in \mathrm{(0,}\,\mathrm{1)}$ with $\alpha  > \mu$.

*If the initial opinion is distributed on [a,b] with expected value E(X*_*0*_) and $d > \frac{1}{2}({\rm{E}}\mathrm{|2}{X}_{0}-a-b|+b-a)$ $(p+\alpha \mathrm{(1}-p{))}^{-1}$*, then with probability 1*, ${{\rm{l}}{\rm{i}}{\rm{m}}}_{t\to \infty }|{X}_{t}(u)-{X}_{t}(v)|=0$ *for all edges* $\{u,v\}\in E$.

Unlike the 1-dimensional case, here we are only able to establish an upper bound for the critical confidence level *d*_*c*_. In fact, as commented in Remark 3.5 in^34^, the case of $D\ge 2$ is much more complicated then the 1-dimensional counterpart and it is even not clear if there exists a critical *d*_*c*_ separating the supercritical and subcritical regimes since the ultimate consensus does not need be monotonic with respect to *d*. Furthermore, we note that the consensus result in Theorem 3 is weaker that in Theorems 1 and 2 (for the supercritical regime) in the sense that only the difference between the opinions of two neighbouring individuals is required to converge towards zero. It is to verify that this is equivalent to the convergence of each individual’s opinion in a finite network. For infinite networks considered in this paper, however, the picture is quite different as one may imagine a situation where the opinion shows wave-like patterns on broader and broader spatial scales with non-vanishing amplitude as time increases.

To prove Theorem 3, we first define the energy of node *u* at time *t* as ${\varepsilon }_{t}(u)=f({X}_{t}(u))$, where $f\,:[a,b]\to \mathrm{[0,}\,\infty )$ is some convex function. Given an edge $e=\{u,v\}\in E$, let *T* be the sequence of arrival times of the Poisson events at *e*. The accumulated energy loss along *e* is defined as

13 $${\varepsilon }_{t}^{\dagger }(e)\,:=\sum _{s\in T\cap \mathrm{[0,}t]}({\varepsilon }_{s-}(u)+{\varepsilon }_{s-}(v)-{\varepsilon }_{s}(u)-{\varepsilon }_{s}(v)),$$

which is nonnegative due to Jensen’s inequality^34^. At time *t*, the total energy of node *u* is defined as ${\varepsilon }_{t}(u)+\frac{1}{2}\sum _{e:e\sim u}{\varepsilon }_{t}^{\dagger }(e)$, where $e\sim u$ means *u* is an end-point of *e*. Following the same argument of [34, Lemma 3.2] and noting that the number of Poisson rings on a single edge in any time period of length *ε* is a Poisson random variable with parameter *λε*, we have the following lemma.

#### Lemma 3.

For any $u\in {{\mathbb{Z}}}^{D}$ and time $t\ge 0$, ${\rm{E}}({\varepsilon }_{t}(u)+\frac{1}{2}\sum _{e:e\sim u}{\varepsilon }_{t}^{\dagger }(e))={\rm{E}}{\varepsilon }_{0}\mathrm{(0)}$.

This means that the total energy at any node is conserved during the opinion exchange process.

#### Lemma 4.

For the above duplex opinion model on ${{\mathbb{Z}}}^{D}$ with $D\ge 2$, $\lambda  > 0$, $\mu \in \mathrm{(0,1/2]}$, and $\alpha ,p\in \mathrm{(0,1)}$. Suppose $\alpha  > \mu$. If $d\in \mathrm{(0,}b-a]$, then with probability 1 for every two neighbours $u,v\in {{\mathbb{Z}}}^{D}$, either $|{X}_{t}(u)-{X}_{t}(v)| > {A}_{t}d$ for all sufficiently large *t* (i.e., $\{u,v\}$ is finally blocked), or ${\mathrm{lim}}_{t\to \infty }|{X}_{t}(u)-{X}_{t}(v)|=0$.

#### Proof.

As commented in^33^, in the following, we will use *A* instead of *A*_*t*_. Choose the energy function $f(x)={x}^{2}$ and fix an edge $e=\{u,v\}$. Let $\delta  > 0$. When there is a Poisson event at *e* at time *t* and *u*,*v* exchange opinions, energy to the amount of $2\mu \mathrm{(1}-\mu )({X}_{t-}(u)-{X}_{t-}(v{))}^{2}$ is lost along the edge; see^33,34^. Hence, if $|{X}_{t-}(u)-{X}_{t-}(v)|\in (\delta ,Ad]$, energy ${\varepsilon }_{t}^{\dagger }(e)$ will increase by the amount of at least $2\mu \mathrm{(1}-\mu ){\delta }^{2}$. Thanks to the memoryless property, given $|{X}_{s}(u)-{X}_{s}(v)|\in (\delta ,Ad]$ at some time $s$, the first Poisson event after time *s* on an edge incident to either *u* or *v* occurs at *e* with probability ${\mathrm{(4}d-\mathrm{1)}}^{-1}$.

In view of the conditional Borel-Cantelli lemma [Corollary 3.2]^36^, this will happen infinitely often with probability 1. If $|{X}_{t}(u)-{X}_{t}(v)|\in (\delta ,Ad]$ at some sufficiently large *t*, then ${\mathrm{lim}}_{t\to \infty }{\varepsilon }_{t}^{\dagger }(e)=\infty$. However, this is impossible since Lemma 3 yields ${\rm{E}}({\varepsilon }_{t}^{\dagger }(e))\le 2{\rm{E}}({\varepsilon }_{0}\mathrm{(0))}\le 2\,{\rm{\max }}\,\{{a}^{2},{b}^{2}\}$. Thereby, with probability 1, for all large enough *t*, $|{X}_{t}(u)-{X}_{t}(v)|\in \mathrm{[0,}\delta ]\cup (Ad,b-a]$.

For small enough $\delta  > 0$, $|{X}_{t}(u)-{X}_{t}(v)|$ cannot jump back and forth between [0, *δ*] and $(Ad,b-a]$ infinitely often. This is because a single Poisson event cannot increase $|{X}_{t}(u)-{X}_{t}(v)|$ by more than *μd*, which for sufficiently small *δ*, is always less than the span of the gap (*δ*, *Ad*) that needs to be crossed due to *μ* < *α*. Since there are only countably many edges, the proof of Lemma 4 is completed.□

#### Proof of Theorem 3.

Fix some $$d\ge (a+b\mathrm{)/2}$$. If $$e=\{u,v\}$$ be a finally blocked edge, then the opinion of node *u* must finally be located in one of the intervals $$[a,b-Ad)$$ or $$(a+Ad,b]$$. It follows from Lemma 4 that this event holds almost surely for any *u* if there are finally blocked edges. Suppose that there is an edge *e* such that

14 $$P(e\,{\rm{is}}\,{\rm{finally}}\,{\rm{blocked}}) > 0.$$

Following a similar argument as in [34, Lemma 3.4], we obtain with probability 1 that $${{\rm{l}}{\rm{i}}{\rm{m}}{\rm{i}}{\rm{n}}{\rm{f}}}_{t\to {\rm{\infty }}}|{X}_{t}(u)$$$$-(a+b\mathrm{)/2|}-a-Ad\ge (a+b\mathrm{)/2}$$ for all $$u\in {{\mathbb{Z}}}^{D}$$.

We choose the energy function $f(x)=|x-(a+b\mathrm{)/2|}$. By Lemma 3 and Fatou’s lemma, we obtain

15 $$\begin{array}{rcl}a+[p+\alpha \mathrm{(1}-p)]d-\frac{a+b}{2} & \le  & {\rm{E}}(\mathop{\mathrm{lim}\,{\rm{\inf }}\,}\limits_{t\to \infty }{\varepsilon }_{t}(u))=E(\mathop{\mathrm{lim}\,{\rm{\inf }}}\limits_{t\to \infty }|{X}_{t}(u)-\frac{a+b}{2}|)\\  & \le  & \mathop{\mathrm{lim}\,{\rm{\inf }}}\limits_{t\to \infty }{\rm{E}}|{X}_{t}(u)-\frac{a+b}{2}|\\  & \le  & \mathop{\mathrm{lim}\,{\rm{\inf }}}\limits_{t\to \infty }{\rm{E}}({\varepsilon }_{t}(u)+\frac{1}{2}\sum _{e:e\sim u}{\varepsilon }_{t}^{\dagger }(e))\\  & \le  & =\,{\rm{E}}({\varepsilon }_{0}(u))={\rm{E}}|{X}_{0}-\frac{a+b}{2}|\mathrm{.}\end{array}$$

Recall that the condition of Theorem 3 implies that ${\rm{d}} > \frac{1}{2}({\rm{E}}\mathrm{|2}{X}_{0}-a-b|+b-a)(p+\alpha \mathrm{(1}-p{))}^{-1}$, which leads to a contradiction. Hence, the assumption (14) must not be true. The proof then follows from applying Lemma 4. □

Theorem 3 can be directly extended to the multiplex setting for a general $\ell \ge 2$.

#### Theorem 4.

(higher-dimensional multiplex networks) *Consider the above continuous opinion model on* ${{\mathbb{Z}}}^{D}$ *with* $D\ge 2$, $\lambda  > 0$, $\mu \in \mathrm{(0,1/2]}$, and ${p}_{i}\in \mathrm{(0,1)}$ for $i=1,\ldots ,\ell$, ${\alpha }_{i}\in \mathrm{(0,1)}$ for $i=2,\ldots ,\ell$ and ${\alpha }_{1}=1$. Suppose ${\alpha }_{i} > \mu$ for all *i*.

If the initial opinion is distributed on [*a*, *b*] with expected value $${\rm{E}}({X}_{0})$$ and $$d > \frac{1}{2}({\rm{E}}\mathrm{|2}{X}_{0}-a-b|+b-a)$$$$(\sum _{i=1}^{\ell }\,{p}_{i}{\alpha }_{i}{)}^{-1}$$, then with probability 1, $${\mathrm{lim}}_{t\to \infty }|{X}_{t}(u)-{X}_{t}(v)|=0$$ for all edges $$\{u,v\}\in E$$.

Some remarks follow: firstly, it is easy to check that the lattice ${{\mathbb{Z}}}^{D}$ in Theorem 4 can be extended to any infinite, locally finite, transitive and amenable connected graph ${G}_{i}=(V,{E}_{i})$ for each $i=\mathrm{1,}\ldots ,\ell$ by using Zygmund’s ergodic theorem; c.f. [34, Remark 3.6]. Recall that a graph is locally finite if every node in it has a finite degree. A graph $G=(V,E)$ is transitive if for any pair of nodes *u* and *v* in it, there is an automorphism $\phi :V\to V$ such that $\phi (v)=u$. A graph $G=(V,E)$ is amenable if there exists a sequence ${S}_{n}\subseteq V$ of finite sets satisfying ${lim}_{n\to \infty }|{\partial }_{E}{S}_{n}|/|{S}_{n}|=0$, where ${\partial }_{E}{S}_{n}$ is the edge boundary of ${S}_{n}$. The following result can be established.

#### Theorem 5.

(general multiplex networks) *Consider the above continuous opinion model, where each layer* ${G}_{i}=(V,{E}_{i})$ *(*$i=1,\ldots ,\ell$*) is an infinite, locally finite, transitive and amenable connected graph. Let* $\lambda  > 0$, $\mu \in (\mathrm{0,}\,\mathrm{1/2}]$, *and* ${p}_{i}\in \mathrm{(0,}\,\mathrm{1)}$ *for* $i=1,\ldots ,\ell$, ${\alpha }_{i}\in \mathrm{(0,}\,\mathrm{1)}$ *for* $i=2,\ldots ,\ell$ *and* ${\alpha }_{1}=1$ *with* ${\alpha }_{i} > \mu$ *for all i*.

*If the initial opinion is distributed on [a,b] with expected value* ${\rm{E}}({X}_{0})$ *and* $d > \frac{1}{2}({\rm{E}}\mathrm{|2}{X}_{0}-a-b|+b-a)(\sum _{i=1}^{\ell }{p}_{i}{\alpha }_{i}{)}^{-1}$*, then with probability 1*, ${{\rm{l}}{\rm{i}}{\rm{m}}}_{t\to \infty }|{X}_{t}(u)-{X}_{t}(v)|=0$ *for all edges* $\{u,v\}\in E$.

Secondly, note that $\frac{1}{2}({\rm{E}}\mathrm{|2}{X}_{0}-a-b|+b-a) < b-a$ unless (i) ${\rm{P}}({X}_{0}\in \{a,b\})=1$ and (ii) *X*_0_ is not constant with probability 1. This indicates that the condition $d > \frac{1}{2}({\rm{E}}\mathrm{|2}{X}_{0}-a-b|+b-a)(\sum _{i\mathrm{=1}}^{\ell }{p}_{i}{\alpha }_{i}{)}^{-1}$ in Theorem 4 stand a good chance to be nontrivial even for multiplex networks in most meaningful situations. Thirdly, we have assumed throughout this paper that the initial opinions following ${\mathcal L} ({X}_{0})$ are i.i.d. However, Theorems 4 and 5 still hold if the initial opinions are stationary and ergodic with respect to the graph automorphisms because no other specific features of i.i.d. variables are used in the above proof. Finally, it seems that agents forming a multiplex network are more difficult to reach consensus for the same reason as remarked for 1-dimensional multiplex networks in the above section. Furthermore, as we have mentioned in the beginning of this section, it is generally even not clear if we can still speak of critical confidence level *d*_*c*_ in *D*-dimensional (*D* > 1) multiplex networks and more general multiplex networks.

### Numerical results

In this section, we conduct agent-based simulations on different finite multiplex networks, including regular ones such as *D*-dimensional lattices which can be viewed as a truncation of ${{\mathbb{Z}}}^{D}$ in Theorem 4, and irregular ones such as small-world and scale-free networks, which obviously violate the regularity conditions in Theorem 5 and are prominent examples of non power-law and power-law networks, respectively. Interestingly, we see that for all such networks, the critical thresholds of consensus tend to agree with the predicted upper bounds in Theorems 4 and 5 in the special cases of uniform *X*_0_ and some choices of Poisson rates associated with the multiple layers.

**Figure 2:**
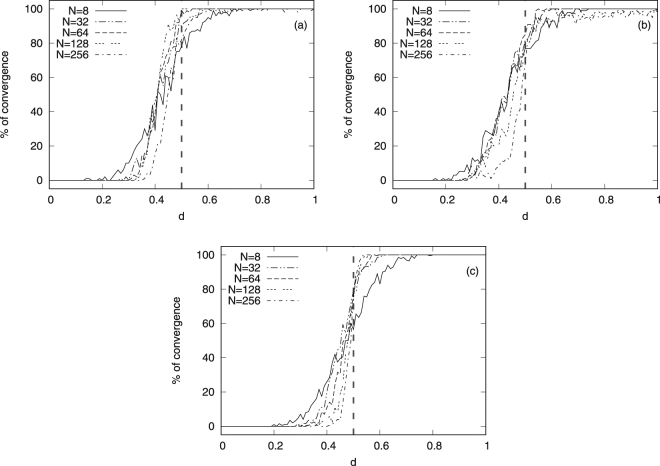
Percentage of opinion-convergence versus *d* for constant *μ* and, different network sizes $N$ and topologies. Panel (a) is for regular networks, panel (b) for Watts-Strogatz (small-world) and panel (c) for Barabási-Albert (scale-free) networks. The vertical dashed line at *d* = 0.5 corresponds to the point around which a sudden jump occurs for increasing network sizes $N$. Note that in these plots we have set the number of layers $\ell$ to 4.

Particularly, in Fig. 2, we plot the percentage of convergence of opinions for five network sizes *N* ranging from 8 to 256 with $\ell =4$ layers each, Poisson rate $\lambda {p}_{i}=0.3$ and $\mu =0.5$ to maximise the convergence rate. At *t* = 0, we initialise each agent $u\in V$ by assigning an opinion value ${X}_{0}(u)\in {\mathbb{R}}$ from the uniform distribution in (0, 1). To check for convergence of opinions, we require that $|{X}_{t}(u)-{X}_{t}(v)| < \hslash ,\,\forall u,v\in G$, where $\hslash ={10}^{-5}$. For each curve, we have run 100 simulations to compute the percentage, each time for a different set of *α* values in Eqs. (1) and (2). Panel (a) shows the results for regular lattices, whereas panel (b) for Watts-Strogatz (small-world) and (c) for Barabási-Albert (scale-free) networks. In all cases, we observe that the system reaches perfect consensus (i.e. 100% opinion convergence) or almost perfect consensus (i.e. >90% opinion convergence), independently of the network structure, and that this starts occurring for different *d* values. It is worth noting however that in all cases, convergence to consensus is reached for *d* > 0.5 denoted by the vertical dashed line in the plots. Particularly, in panel (a), for *N* = 8, the percentage of convergence starts to increase from very small *d* values with the tendency to increase as *N* increases, for example for *N* = 8, it starts at $d\approx 0.25$ whereas for *N* = 256 at $d\approx 0.37$. Surprisingly, the jump from very small (almost 0%) to very big (almost 100%) percentage of opinion convergence (reach of consensus) occurs at *d* = 0.5. Similar conclusions can be drawn for the Watts-Strogatz (small-world) networks in panel (b) and Barabási-Albert (scale-free) networks in panel (c). This is reminiscent of a first order phase-transition as a function of *d* (order parameter) that might exist for infinitely big network sizes (i.e. $N\to \infty$) and is an open question.

Note that the convergence parameter *μ* considered above is constant and independent of the node-degrees and thus, assumes that all agents have the same paces towards adjusting their opinions. As this is rather ideal and not in agreement with real-life situations, we consider in the following two archetypal variants given by

16 $${\mu }^{+}(u,v)=\frac{{\rm{\deg }}(u){\rm{\deg }}(v)}{2\,\mathop{{\rm{\max }}}\limits_{u\sim v}\{{\rm{\deg }}(u){\rm{\deg }}(v)\}}$$

and

17 $${\mu }^{-}(u,v)=\frac{\mathop{{\rm{\min }}}\limits_{u\sim v}\{{\rm{\deg }}(u){\rm{\deg }}(v)\}}{2\,{\rm{\deg }}(u){\rm{\deg }}(v)},$$

featuring the degree-centrality dependent scenarios, where the max and min of ${\rm{\deg }}(u){\rm{\deg }}(v)$ is computed among all edges *u* adjacent to *v* (i.e. $u\sim v$). Namely, we replace the convergence parameter *μ* in Eqs (1) and (2) by ${\mu }^{+}(u,v)$ indicating that higher-degree nodes are more willing to adjust their opinions (and ${\mu }^{-}(u,v)$ indicating the opposite way). Clearly, ${\mu }^{+}(u,v)$ and ${\mu }^{-}(u,v)$ are within the interval (0, 0.5] for connected networks. *μ*^+^ is close to 0.5 for a well-connected pair of nodes, whereas *μ*^−^ is close to 0.5 for a poorly-connected pair of nodes. Our choice of degree-related convergence parameter here naturally reflects the idea of the number of neighbors/contacts in social networks, and models the possible mechanisms of heterogeneous psychological, habitual and cultural backgrounds in opinion spreading. Although degree-centrality apparently depends on the structure of the layers of the multiplex network, our numerical results indicate that the convergence parameters do not affect the ultimate opinion configuration. It is worth mentioning that there are other measures of centrality as well, such as eigenvector-like centralities^37^ and measures based on random walks^38^, that have been studied in the context of multiplex networks. However, as commented above, it is reasonable to expect similar results for other convergence parameters mediated by more complicated measures.

**Figure 3:**
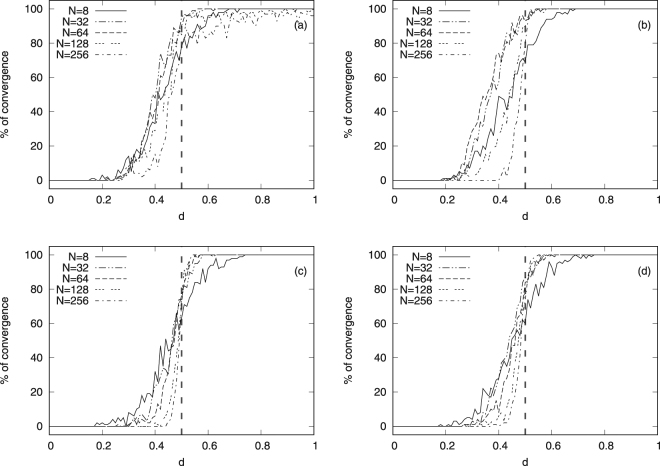
Percentage of opinion-convergence versus *d* for different network sizes *N* and topologies using the degree-dependent convergence parameters ${\mu }^{+}(u,v)$ and ${\mu }^{-}(u,v)$ in Eqs (1) and (2). Panels (a) and (b) are for Watts-Strogatz (small-world) networks with ${\mu }^{+}(u,v)$ and ${\mu }^{-}(u,v)$, respectively and panels (c) and (d) for Barabási-Albert (scale-free) networks with ${\mu }^{+}(u,v)$ and ${\mu }^{-}(u,v)$, respectively. The vertical dashed line at *d* = 0.5 corresponds to the point around which a sudden jump occurs for increasing network sizes $N$. Note that in these plots we have set the number of layers $\ell$ to 4.

The simulation results for the degree-centrality dependent parameters *μ*^+^ and *μ*^−^ are presented in Fig. 3, where we have used the same network sizes, number of layers, Poisson rate and number of simulations as in Fig. 2 to compute the percentage of convergence of the opinions. In particular, panels (a) and (b) are for Watts-Strogatz (small-world) networks with *μ*^+^(*u*, *v*) and *μ*^−^(*u*, *v*), respectively and panels (c) and (d) for Barabási-Albert (scale-free) networks with *μ*^+^(*u*, *v*) and *μ*^−^(*u*, *v*), respectively. It is found that these degree-dependent convergence parameters (i.e. *μ*^+^(*u*, *v*) and *μ*^−^(*u*, *v*)) do not alter the ultimate opinion configurations and that the confidence threshold still remains. This is in line with previous findings for Deffuant models in the case of single-layer networks^8,19^ and our results extend this finding to multiplex networks.

## Discussion

In this paper, we studied analytically and numerically opinion dynamics over multiplex networks with an arbitrary number of layers, where the agents interact with each other with bounded confidence. In the literature, agents are generally assumed to have a homogeneous confidence bound and here we sought to study analytically and numerically opinion evolution over multiplex networks with respective confidence thresholds and general initial opinion distributions. We explicitly identified the critical thresholds at which a phase transition in the long-term consensus behaviour occurs. We then discussed the interaction topology of the agents by using multiplex *D*-dimensional lattices and extended them to general multiplex networks under some regularity conditions. Our results reveal the quantitative relation between the critical threshold and initial distribution. We also performed numerical simulations and illustrated the consensus behaviour of the agents in regular lattices and, small-world and scale-free networks. We found that the numerical results agree with our theoretical ones and in particular, the critical thresholds of consensus tend to agree with the predicted upper bounds in Theorems 4 and 5 for all network topologies considered in the special cases of uniform *X*_0_ and some choices of Poisson rates associated with the multiple layers.

Moreover, we used the Deffuant opinion model represented as a stochastic process for the evolution of opinions that includes heterogenous confidence bounds and features general initial distributions and, determined the critical threshold by employing probability methods. The main results of our work are Theorems 2 (for *D* = 1) and Theorem 4 (for *D* > 1) which extend previous results in^33,34^ by considering both multiplex structures with $\ell  > 1$ and general initial opinion distribution ${\mathcal L} ({X}_{0})$. We show that both the initial distribution and multiplex structure play an important role in the phase transition of opinion evolution in an infinite *D*-dimensional regular lattice in the sense that the critical confidence bound in the case of Theorem 2 (or an upper bound of it in the case of Theorem 4) is influenced by both factors. Our results indicate that multiplexity hinders consensus formation when the initial opinion configuration is within a bounded range. This is numerically found to be true in more general networks including small-world and scale-free networks, which are ubiquitous in the real world. Our results provide insight into information diffusion and social dynamics in multiplex real-life systems modeled by networks. However, the theoretical proof of this is beyond the scope of this paper as it would require the development of substantially new techniques that we leave for a future publication.

It is worth mentioning that the networks considered here are static, and thus the connectivity remains fixed throughout opinion spreading. As a result, structural properties such as centrality, correlations, homophily, and assortativity, remain the same throughout opinion spreading. On the other hand, in networks of human social interactions, the interaction can be assorted according to, e.g., the channels used for communication such as face-to-face, mobile phone, and social network services^39^. Certain social mechanisms such as assortativity and homophily (namely, the tendency of individuals to align to behaviours of their friends) are popular in real social networks and may play a key role in opinion formation and its dynamics. For instance, it is shown in^40^ that the higher the homophily between individuals in a multiplex network, the quicker is the convergence towards cooperation in the social dilemma. Multiplex social ecological network analysis unravels that node heterogeneity has a critical effect on community robustness^41^. However, as far as convergence of the opinion spreading is concerned, our numerical results, for three different characteristic types of multiplex networks (regular, small-world and scale-free), indicate the same qualitative and almost similar qualitative tendency to reach consensus as a function of *d* for different network architectures. This is in agreement with our theoretical results. In fact, assortativity and homophily are not included in our theoretical analysis because they usually subvert the transitivity and amenability conditions (see Theorem 5) that form the foundation of our mathematical technique. In a future work, we will focus on how to incorporate multiplex characterisations by means of structural measures, such as homophily and assortativity of the multiplex network, into analytically tractable opinion-formation models. Finally, temporal or co-evolving networks with random environments also seem appealing in this respect as they might lead to differences with respect to convergence to consensus.
